# A Novel Estimator for the Rate of Information Transfer by Continuous Signals

**DOI:** 10.1371/journal.pone.0018792

**Published:** 2011-04-11

**Authors:** Jouni Takalo, Irina Ignatova, Matti Weckström, Mikko Vähäsöyrinki

**Affiliations:** Department of Physics, Biophysics and Biocenter Oulu, University of Oulu, Oulun yliopisto, Finland; Lund University, Sweden

## Abstract

The information transfer rate provides an objective and rigorous way to quantify how much information is being transmitted through a communications channel whose input and output consist of time-varying signals. However, current estimators of information content in continuous signals are typically based on assumptions about the system's linearity and signal statistics, or they require prohibitive amounts of data. Here we present a novel information rate estimator without these limitations that is also optimized for computational efficiency. We validate the method with a simulated Gaussian information channel and demonstrate its performance with two example applications. Information transfer between the input and output signals of a nonlinear system is analyzed using a sensory receptor neuron as the model system. Then, a climate data set is analyzed to demonstrate that the method can be applied to a system based on two outputs generated by interrelated random processes. These analyses also demonstrate that the new method offers consistent performance in situations where classical methods fail. In addition to these examples, the method is applicable to a wide range of continuous time series commonly observed in the natural sciences, economics and engineering.

## Introduction

Shannon's classical information theory [1] has been widely applied to the fields of engineering and natural sciences. Instead of a general measure of information transfer, the information capacity of a Gaussian channel (later referred to as information capacity) is the most often used estimator for analyzing information processing. However, its use assumes that the input has Gaussian statistics, that the system is linear and time-invariant, and that any noise in the data is Gaussian and additive [2]. Here we introduce a novel information rate estimator that only requires assumptions of stationarity and ergodicity. It works with a single realization of the signals of experimentally realistic duration. Practical implementation of the estimator is further facilitated by signal conditioning methods that increase computational efficiency.

## Results

### Derivation of the method

We start by deriving an auxiliary equation for the random processes. A random process, 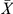, can be formulated as a time indexed sequence of random variables, X_i_, as 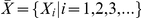. By assuming that 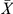 is stationary and ergodic the entropy rate of the random process, 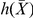, can be defined as:
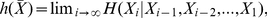
(1)where 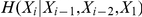 is the conditional entropy, which follows the general definition for two random variables X and Y, 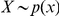 and 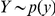, respectively:
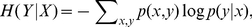
(2)where p(x,y) is the joint probability distribution and p(y|x) is the conditional probability distribution. The entropy rate is a measure of how fast entropy of the random process increases at the limit of infinitely long random processes. To circumvent the requirement for infinitely long samples of the random processes, we approximate them as a Markov process of order L. This means that the random process generating the data works in such a way that any data point only depends on L points in the past but no further. Following this approximation, the conditional state of X_i_ is independent of states beyond X_i-L_ and the following holds for the probability distribution:
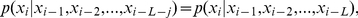
(3)where 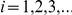 and 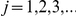 (j is an index parameter related to the Markov assumption). Using this formulation the entropy rate in equation (1) can be presented as:
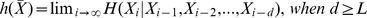
(4)and d is defined as 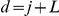. By using the general chain rule of entropy
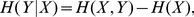
(5)where 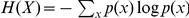 and 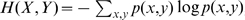, and equation (4), the auxiliary equation for the entropy rate can be written as:
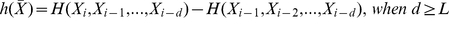
(6)


**Figure 1:**
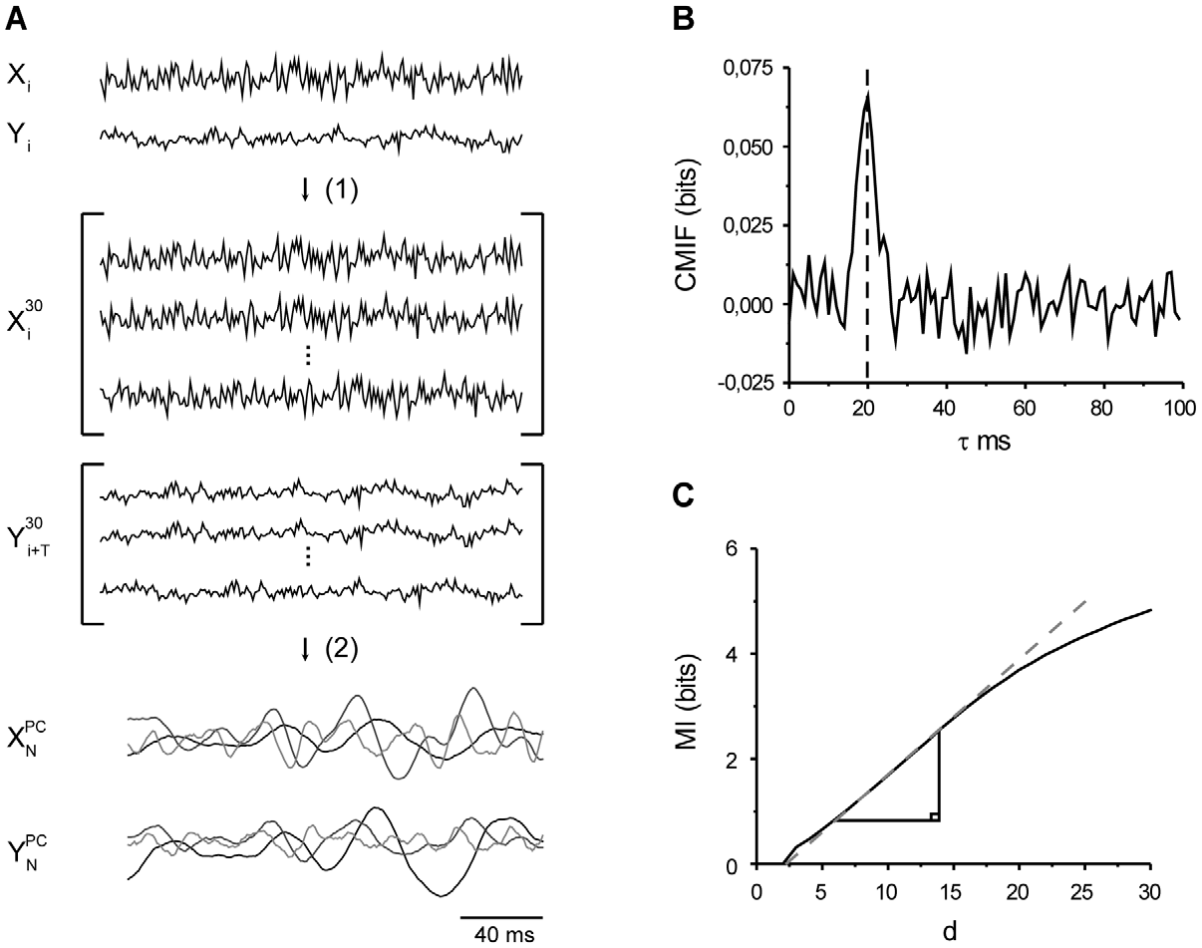
Illustration of the steps involved in the information rate estimation. **A**, Sections of simulated input, X, and output, Y; (1) input and time-delayed output with d = 30; (2) the three largest principal components used for the MI estimate. **B**, CMIF with T = 20 ms. **C**, MI as a function of d; information rate of 220 bits/s was obtained from the linear fit (grey). Graph corresponds to the 4^th^ data point in Figure 2.

The information rate, R, between two random processes 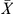 and 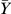 quantifies the rate of increase in the mutual dependencies between the processes (i.e. it provides a dynamic measure of information transmission). 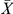 could be the input and 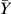 the output of a random process, or they could be two outcomes of interrelated random processes. The information rate can be defined as [3]:
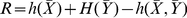
(7)Assuming that 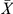 and 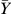 are Markov processes and that they are also jointly a Markov process of order L, allows writing the probability distribution as:
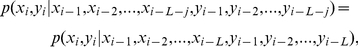
(8)where 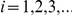 and 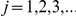 The information rate of equation (7) can be reformulated using equation (6) and the Markov assumptions in equations (3) and (8) as:
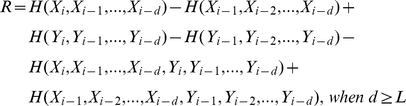
(9)Below, a definition of mutual information (MI), a measure of mutual dependence between two random variables is used (i.e. a static measure of the information transmission). MI, I(X,Y), can be defined as:
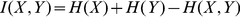
(10)where the MI between the two random variables X and Y is:
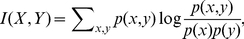
(11)allowing a reformulation of equation (9) to give the information rate R as:
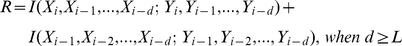
(12)This recursive equation can be solved with respect to the MI, as a linear function of d:
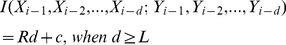
(13)In this equation 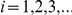 and c is an additional constant that depends on the initial conditions. In practical terms, samples of some tens of thousands to a hundred thousand data points are required from the two simultaneously measured signals (i.e. outcomes of the random processes). Then the information rate can be estimated from the linear slope of the obtained MI values as d is increased (see Figure 1a for practical illustration of the d parameter).

Several algorithms exist to estimate the MI between two random variables. However, in the case of continuously distributed random processes, estimation of MI through direct application or binning of the probability density could produce severely biased results [4]. Therefore, we selected a k-nearest neighborhood algorithm for estimating the MI, which is also known to handle multivariate MI adequately [4], [5]. However, according to the original publication of the MI algorithm [5], d is expected to be limited to values below 5 with practical data sets. This was found to be insufficient in preliminary testing of the estimator. Therefore, we introduce two signal conditioning methods to circumvent this limitation as next steps in the derivation of the estimator.

Significant lags arise in physical systems, which mean that information from one signal may only appear in the other after a delay. Estimating MI does not require sampling the random processes in any specific time progression (assuming d≥L) and it is, therefore advantageous to arrange the analysis to use the most mutually dependent samples. The degree of mutual dependence can be measured by the cross MI function CMIF [6]:
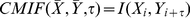
(14)where 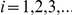 and τ is the delay between the two processes. The CMIF is closely related to the more familiar cross-correlation function but, instead of the linear dependencies, it estimates the mutual dependencies. Maximum CMIF occurs at mean latency, T, which is introduced to the MI estimates as: 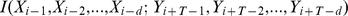, i.e. it shifts the latter dataset (Y) by T points forward in time. This also changes the joint probability distribution accordingly:
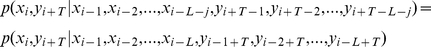
(15)

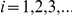
 and 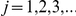 Equation (15) is satisfied at smaller L than equation (8), which means that the information rate estimate is obtained with smaller d. Therefore, introduction of a system-dependent delay to the estimator will make it converge faster as a function of d.

As a second signal conditioning method, principal component analysis (PCA) can be used to focus analysis on the most significant features of the high-dimensional samples of the two signals (dimensionality is defined by d). In PCA a data set is analyzed to find orthogonally most independent sets of components in the order of variance in the data that they can account for. An additional benefit of using the PCA is that practical data sets are always contaminated by noise, which can be at least partially filtered out by PCA without changing the information rate estimate. The PCA is first applied to the second signal, time shifted by T, as described above. The N highest principal components (PCs), 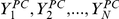, are selected and multiplied with the first signal to give the corresponding N highest PCs, 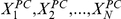. As a result, the dimensionality of the original two signals is reduced from d to N. It should be noted that all PCs with significant contribution to the original random processes should be included in the estimator to avoid biased results. In practice, we have found it sufficient to use 2–4 PCs, which in our data sets corresponded to over 98% of the eigenvalues.

The time-shifted and dimensionally reduced signals are introduced to equation (13) to give the final formulation of the information rate estimator:
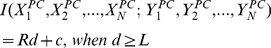
(16)In principle, it is possible to estimate the information rate, based on the MI, with just two different d values, but more should be used to decrease the statistical error of the estimate. Using the base-2 logarithm for the MI calculation gives units of bits/transmission, which can be converted to time-dependent measure of bits/s when the interval between the two sample points of the signals is known. The major steps of the novel estimator are illustrated in the Figure 1 (see also Materials and Methods).

### Validation of the method

**Figure 2:**
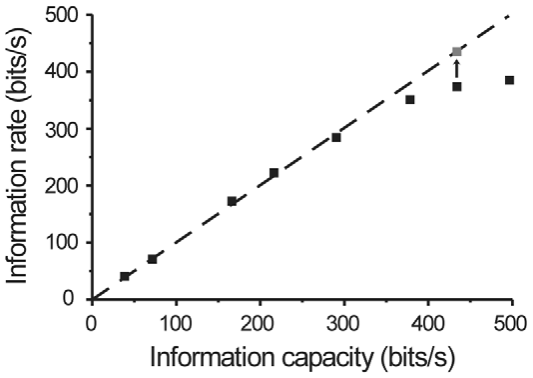
Information rate vs. information capacity of a Gaussian channel. Grey square corresponds to the estimate from 600000 data points.

To validate the novel estimator, a data set was simulated that meets the assumptions of the Shannon's information capacity estimate, allowing the results from the novel estimator to be compared with a known, valid estimate of information capacity. Input x(t) was generated as a random process of 40000 normally distributed data points with time difference of 1 ms between the points (Figure 1A). The input was filtered by a low pass filter with coefficients of a(i), where i = 0,1,…40, to generate output
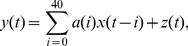
(14)where z(t) indicates linearly added Gaussian noise with variance ranging from 0.0025 to 2.26 (Figure 1A). This produced simulated test data with varying information rates that could readily be computed. The information rate estimated by the novel method was found to match the information capacity of the Gaussian channel up to about 360 bits/s, after which the novel estimate started to plateau at an underestimate (Figure 2). This deviation is attributable to the MI estimator algorithm, which requires ever longer samples for data at higher MI values. Additional analysis showed that a sample size of 600000 points is required to achieve an accurate information rate estimate at 430 bits/s (Figure 2). However, it should be noted that this problem is alleviated with real data sets. With correlated signals the k-nearest neighbor statistics of the MI algorithm [5] can be estimated reliably with smaller sample sizes and the computational efficiency is also improved as the number of the required PCs is significantly lower than with the uncorrelated, random signals.

### Example applications

**Figure 3:**
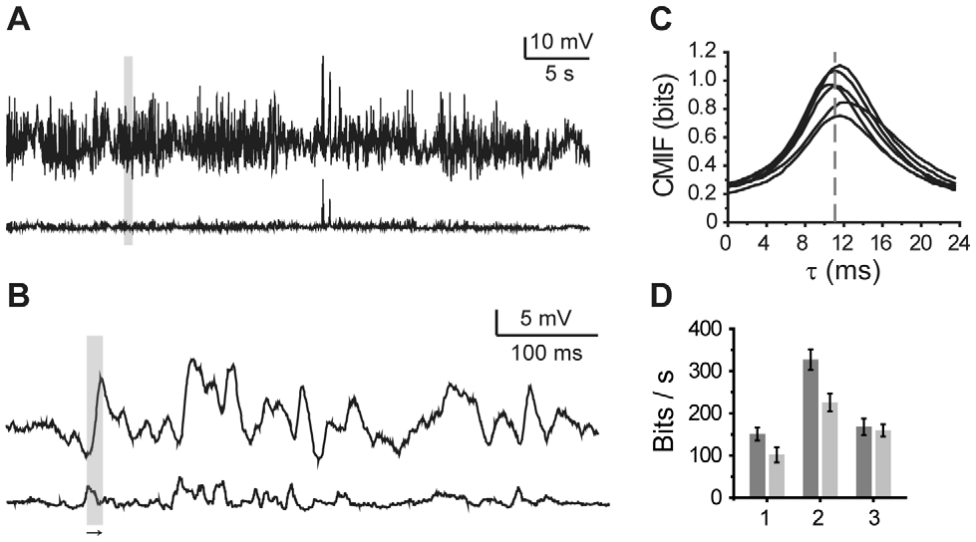
Information processing in blowfly photoreceptors. **A**, An example of the photoreceptor response (top) to the light stimulus (below; stimulus 2 in **D**). **B**, Enlargement of the section marked with the grey box in **A**; output follows the input with a delay of ca. 11 ms (grey box). **C**, CMIFs from six photoreceptors; delay observed in **B** is marked with the grey dashed line. **D**, Mean ± s.d. of the information rates (grey) and information capacities (light grey) for three different stimulus sequences (significantly different in each case; Student's t-test; (1) p<0.00031, (2) p<0.000011 and (3) p<0.048).

Two example applications, visual sensory neuron signals and surface temperature measurements from climate data, were selected to demonstrate the general applicability of the novel estimator. Blowfly photoreceptors were used to illustrate information processing by a nonlinear system through input-output analysis. This is a well established model system where the dynamic light input is efficiently and reliably encoded into a graded membrane voltage response of limited amplitude range. A majority of previous studies have relied on information capacity estimates obtained by white noise stimulation [7]–[11]. However, the statistics of naturally occurring inputs are non-Gaussian and correlated in time, giving highly nonlinear responses [12]–[15]. We recorded voltage responses from six photoreceptors to three different sequences of naturalistically varying light intensity stimuli, selected randomly from a published data set [12] (Figure 3A and B). The CMIF between the stimulus and the responses indicated that a lag of approximately 11 ms existed in the system (Figure 3C). Depending on the specific stimulus sequence used, the information rate was found to vary from 152 bits/s to 332 bits/s. The information capacity estimates for the same data set ranged from 102 bits/s to 226 bits/s (Figure 3D). The significant underestimation by information capacity analysis is attributable to the failure of the data to meet the required assumptions. This underlines the importance of using an accurate estimator in this and similar cases of nonlinear processing. The observed strong dependence of information rate on the specific naturalistic stimulus sequence is also an interesting finding that deserves more careful investigation in future work.

**Figure 4:**
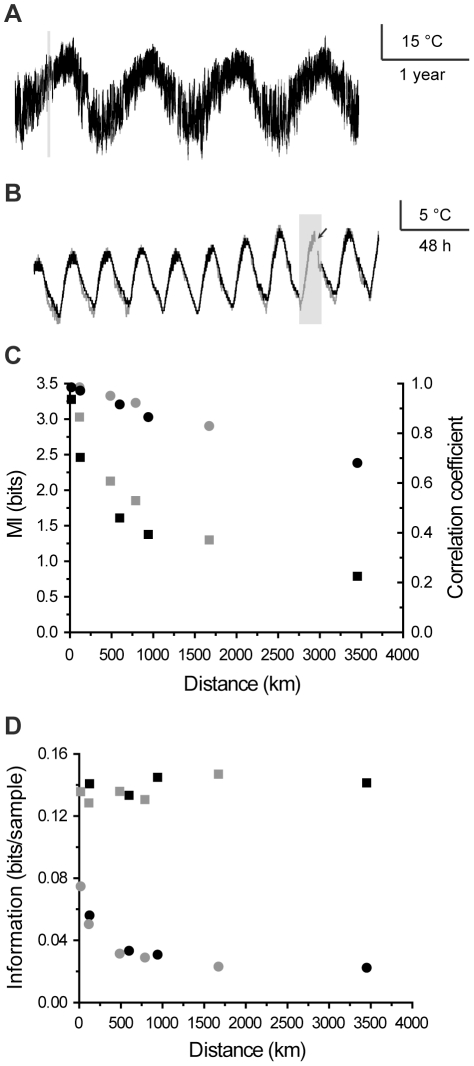
Information theoretical analysis of the climate data. **A**, 4 years of surface temperature data from two nearby weather stations in Dallas, Texas. **B**, 10 day enlargement of the section indicated with the grey bar in **A**. Data points are missing on the highlighted section on either one or both of the traces (indicated by arrow). **C**, MI (squares) and linear correlation coefficients (circles) as a function of distance between the observation points. Latitudinal (black) and longitudinal (grey) data sets span across the continent orthogonally. **D**, Information rate (squares) and capacity (circles) as a function of distance. The color coding is as in **C**.

**Figure 5:**
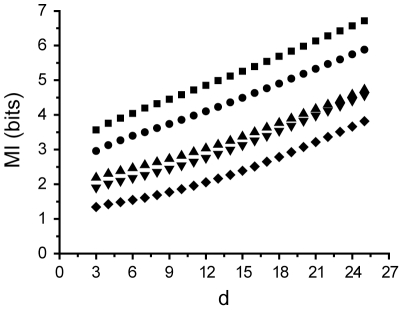
MI as a function of d for the weather stations with varying latitudinal distance.

Surface temperature measurements from US weather stations with varying distances from each other either by latitude (from San Diego, CA to Charleston, SC) or longitude (from Dallas, TX to Grand Forks, ND) were retrieved from a climate data set (Table 1; see Methods). From a systems analysis point of view, these measurements can be considered as readouts of interrelated processes that share a common input. Information theory has been previously applied to climate data to estimate the quality of weather forecasts [16], [17], but we are not aware of reports where the information transfer rate between any two observation points have been analyzed. Temperature data showed clear seasonal (Figure 4A) and daily rhythms (Figure 4B). The lag between the two observation sites was quantified with the CMIF and varied from 10 to 50 minutes without any apparent correlation with distance. Therefore, the variability is likely to result from the latitudinal variation of the weather stations within a time zone, which results in a variable delay of the sun-dependent ambient temperature cycle. Linear correlation coefficients were also calculated from the data and they were found to decrease with a linear trend as the distance between observation points increased (Figure 4C). This finding matches results of earlier work [18]. However, the MI that captures all the dependencies instead of just the linear ones was found to decrease with much faster trend (Figure 4C), suggesting that previous estimates of the dependencies were significantly underestimated. Continuing, information rates estimates showed no variation with distance (Figure 4D). This contrasts with information capacity estimates that were most likely to fail because of strong nonlinear dependencies between any two datasets (Figure 4D). These consistent information rates suggests that, although MI varies as a function of distance, the average amount of new information added over time is independent of distance between observation points (i.e. the slope of the MI(d) curve is constant, see Figure 5). This could be interpreted as meaning that despite the local variation of the nonlinear transformation of common inputs (e.g. sunlight) into a surface temperature, the temporal dynamics of the underlying climate process is similar across the observation sites. However, more detailed analysis and interpretation of the data are beyond the scope of this work.

**Table 1:** US Weather stations used in the analysis.

Station 1	Station 2	Distance (km)
Latitudinal variation		
Dallas Fort Worth International Airport, TX	Dallas Love Field, TX	18
Evers International Airport, MS	Meridian Key Field Asos, MS	125
Charleston Air Force Base-International Airport, SC	Montgomery Regional Airport, AL	599
Evers International Airport, MS	Charleston Air Force Base-International Airport, SC	942
San Diego International Airport, CA	Charleston Air Force Base-International Airport, SC	3453
Longitudinal variation		
Dallas Fort Worth International Airport, TX	Dallas Love Field, TX	18
Grand Forks International Airport, ND	Hector International Arpt, ND	117
Grand Forks International Airport, ND	Sioux Falls/foss Fi, SD	487
Grand Forks International Airport, ND	Lincoln Municipal Airport, NE	790
Grand Forks International Airport, ND	Dallas Fort Worth International Airport, TX	1674

## Discussion

We have presented a novel method of estimating the information rate between two continuous signals. Unlike the popular information capacity estimator, our method does not restrict the signal and noise statistics or require linearity. Importantly, it is also practical to implement and computationally efficient.

An alternative information rate estimator was recently introduced for continuous signals, based on varying digitization levels and extrapolation to infinite data size, number of signal levels and sampling rate [15]. In contrast to the present estimator, it depends on reliable estimates of the entropy rate of the signal and the noise in the output, requiring data sets with numerous input-output realizations. This limits its use to studies where the input can be controlled by the investigator, which is not possible for data such as the climate records analyzed here.

Although the main purpose of the example applications was to demonstrate the performance of the estimator, some interesting preliminary results about the two applications were also obtained. Photoreceptor information rate was found to vary strongly with the specific stimulus sequence (Figure 3D). Although varying mean brightness of the stimuli might explain part of this phenomenon (dimmer stimuli inherently contain larger shot noise), even the approximately equally bright stimulus sequences gave significantly different information rates. This suggests that the system is better tuned to some stimuli than to others, which is an interesting topic for future studies. With the climate data set, MI was more strongly dependent on distance than the linear correlation coefficient would indicate. This suggests that nonlinear transformations of the sunlight-to-surface temperature and/or the extrinsic influences become increasingly different at longer physical separations. In contrast, information rate was found to be constant, suggesting that surface temperature results from dynamic processes that integrate past events in a similar manner at different observation sites.

In conclusion, we anticipate that the presented estimator could become a powerful analysis tool in applications where information theoretical analysis has not been previously possible. In biomedical research, it could be applied to the analysis of signals such as those obtained by MRI imaging or to fluorescence signals from cellular dye indicators. Emerging applications also include analysis of biochemical networks [19] and control of gene expression [20]. In general, the method is widely applicable to analysis of continuous time series commonly studied in the natural sciences, economics and engineering.

## Materials and Methods

### Specification guide for the estimator parameters

**Figure 6:**
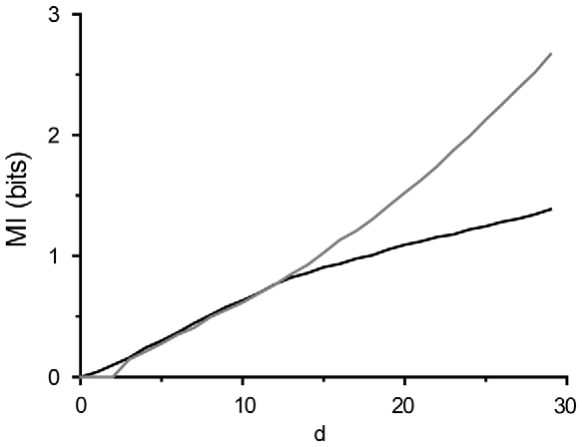
N-parameter selection. No clear linear region is observed if too small N is used in the estimator (black), which would result in an underestimate; too large N gives rise to an erroneous linear range appearing with large d and fitting that region would lead to an overestimate (grey).

Parameters used in the information rate estimator are presented in the Table 2. A Matlab® implementation of the method is available at the authors' website (http://www.physics.oulu.fi/bons/). The recipe for using the estimator can be summarized as follows:

1. **Specify input and output signals.** At least 10000 points are required for the multivariate statistics; increasing n decreases the statistical error and allows reliable estimates of high MI.
2. **Determine T (the time-lag between two data sets).** T is automatically determined using the CMIF. Its exact value is not critical but poor estimates may result in an increased L.
3. **Specify N (number of PCA components).** N should be selected so that over 98% of the variance is explained by the PCA eigenvalues (this restriction may be loosened for noisy signals). High MI requires large N. It should be noted that too small N leads to an underestimated information rate and, on the other hand, too large N may give rise to a second erroneous linear region leading to an overestimate (Figure 6).
4. **Select range for the linear fit (in the MI-plot).** L should be used as the lower limit for the d-values included in the fit (d≥N). L can be estimated from the point when the MI curve becomes linear. If the auto-correlation of the input signal is zero, L is the half-width of the CMIF peak. Maximum d-values used for the fit should be limited to the point where MI starts to deviate from the linearity (d≤T). Note that after the maximum value is exceeded, the MI estimates become underestimated and the slope of the curve reduces until it levels off at zero.
5. **Adjust k (the number of nearest-neighbors in the MI computation algorithm).** The value of k should be adjusted so that the linear part of the curve becomes well defined. A value of unity produces too much statistical error (MI values are scattered around the line) and too large k leads to an underestimate. High MI requires small k, and in practice values between 2 and 6 have provided good results.

**Table 2:** Parameters used in information rate estimator.

Parameter	Definition	Exemplary values*
n	no. of data points	40000
k	no. of nearest neighbors used in MI estimator	3
T	delay between two signals	20
N	no. of PCA components used in MI estimator	3
L	order of the Markov process	10
d	dimension of the random process (variable in information rate estimator)	10–14 (used for linear fit)

*Example values are for the data shown in Figure 1.

### Information capacity estimate

The information capacity was estimated using the coherence function
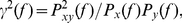
(21)where P_x_(f) and P_y_(f) are power spectrum of the input and output, and P_xy_(f) their cross power spectrum, respectively. The information capacity, C, can be estimated as:
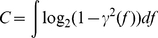
(22)


### Electrophysiology

Female blowflies (*Calliphora vicina*) were used in the experiments according to well-described preparation methods and using a previously reported experimental setup [8], [11], [15]. Photoreceptor voltage responses were recorded intracellularly with aluminosilicate microelectrodes manufactured with a laser puller (P-2000; Sutter Instrument, USA) and filled with 2 M K-acetate solution (electrode resistances 100–130 MΩ). Signals were amplified with an intracellular amplifier (SEC-05L; NPI, Germany) and recorded with a computer controlled data acquisition system with a sampling frequency of 2.4 kHz (DAQ-board: PCIe-6259, National Instruments, USA; custom Matlab® software). Light stimulus was always aligned with the photoreceptor's optical axis and a photoreceptor was accepted to the data set if it had a resting potential below −55 mV, input resistance higher than 25 MΩ and a maximum response to a bright flash of light larger than 50 mV.

### Climate data

Climate data recorded at weather stations across the USA with 1 minute intervals was downloaded from the National Environmental Satellite, Data and Information Service (http://www.ncdc.noaa.gov/oa/climate/climatedata.html#asosminutedata). The weather stations used in the analysis were selected along orthogonal lines near latitude 33° and longitude −97° (Table 1).

**Figure 7:**
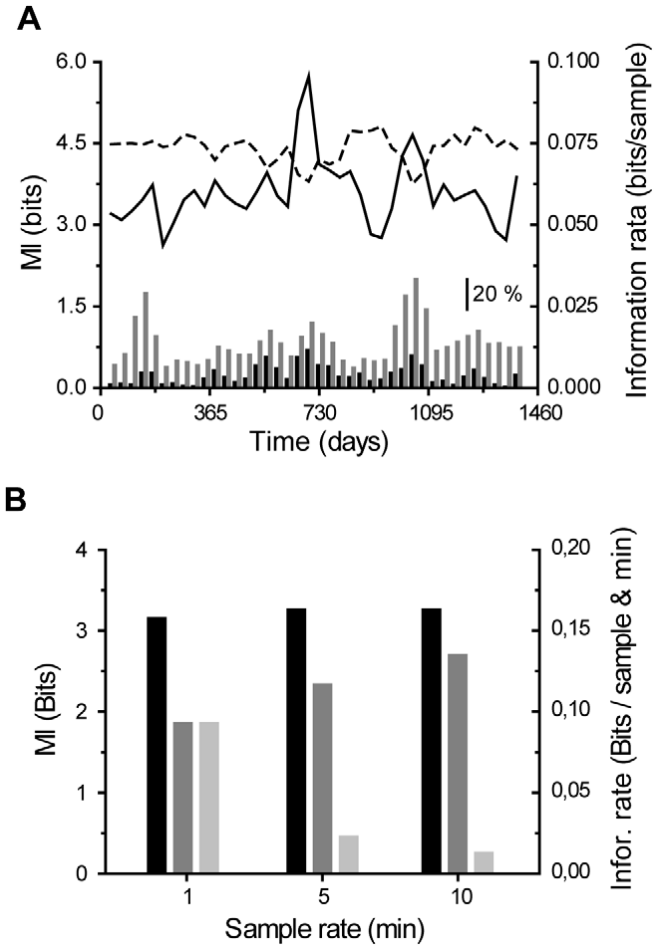
Climate data preprocessing. **A**, MI and information rate (dashed line) over four years of data; inset shows the fraction of missing data points occurring on either one (grey) or on both data series simultaneously (black). **B**, MI (black), information rate per sample (dark grey) or per minute (light grey) of 100,000 data points with different sample intervals.

Missing data points were commonly encountered in the data sets (Figure 4A and 7A) and they were especially numerous for the weather stations in Dallas, TX (Table 1; Figure 4A). Interpolation was used to make the data continuous to enable estimation of bit rates per time unit. The piecewise cubic Hermite polynomial method was used to interpolate the missing data points (Figure 7A). Further analysis of these Dallas observation sites was done for 4 years of data using a sliding window of 100,000 minutes, each window overlapping 50% with the preceding one. It can be clearly seen that both the MI and the information rate are relatively constant over time, except for the sections where large fractions of the data consist of missing data points (Figure 7A). This is especially pronounced in sections where the data was simultaneously missing from both observation sites and where the data consists of smooth curves generated by the interpolating algorithm (Figure 7A). As a result the estimate of the MI was erroneously increased and the estimate of the information rate was decreased. However, it should be emphasized that this analysis represents the worst case scenario of the used data set. In addition, the two first years of data were used in the later analysis, which precludes most of the section with largest errors of the illustrated data. Therefore, we conclude that the errors due to the missing data points and/or interpolation do not change the general validity of the results presented in Figure 4.

To enable analysis of the data sets of several years in duration, data was down-sampled with an anti-aliasing FIR filter and a Kaiser window into 10 minute sample intervals. 100,000 data points were used in the analysis corresponding to two years in time (years 2000 & 2001 in the data set no. 6406). The effect of the down-sampling on the estimates was studied. Sample intervals of 1, 5 and 10 minutes were used and 100,000 samples were analyzed in each case. The MI estimates were found to be unaffected, but the information rate per sample increased slightly with the longer sample intervals (Figure 7B). The original data includes rather large discretization noise (temperature was measured with one degree intervals) and the reduction of this noise attributable to the interpolation could explain the observed increase in the information rates. The information rate per time unit decreased as expected, because the same amount of information was divided by a larger time unit (as a universal law the amount of information cannot be increased by any subsequent processing). Although down-sampling affects the absolute bit rate values the error is systematic and does not affect the results of the comparative analysis made between the observation sites (Figure 4).
